# A study protocol for the evaluation of occupational mutagenic/carcinogenic risks in subjects exposed to antineoplastic drugs: a multicentric project

**DOI:** 10.1186/1471-2458-11-195

**Published:** 2011-03-30

**Authors:** Massimo Moretti, Roberta Bonfiglioli, Donatella Feretti, Sofia Pavanello, Francesca Mussi, Maria G Grollino, Milena Villarini, Anna Barbieri, Elisabetta Ceretti, Mariella Carrieri, Annamaria Buschini, Massimo Appolloni, Luca Dominici, Laura Sabatini, Umberto Gelatti, Giovanni B Bartolucci, Paola Poli, Laura Stronati, Giuseppe Mastrangelo, Silvano Monarca

**Affiliations:** 1 Department of Medical-Surgical Specialties and Public Health, University of Perugia, Via del Giochetto, 06122 Perugia, Italy; 2 Department of Internal Medicine, Geriatrics and Nephrology, Section of Occupational Medicine - Alma Mater Studiorum, Sant'Orsola-Malpighi Hospital, University of Bologna, Via Palagi 9, 40138 Bologna, Italy; 3 Department of Experimental and Applied Medicine, Hygiene Section, University of Brescia, Viale Europa 11, 25123 Brescia, Italy; 4 Department of Environmental Medicine and Public Health, University of Padova, Via Giustiniani 2, 35128 Padova, Italy; 5 Department of Genetics, Biology of Microrganisms, Anthropology, Evolution, University of Parma, Parco Area delle Scienze 11A, 43124 Parma, Italy; 6 Unit of Radiation Biology and Human Health, ENEA CR Casaccia, Via Anguillarese 301, 00123 Rome, Italy

## Abstract

**Background:**

Some industrial hygiene studies have assessed occupational exposure to antineoplastic drugs; other epidemiological investigations have detected various toxicological effects in exposure groups labeled with the job title. In no research has the same population been studied both environmentally and epidemiologically. The protocol of the epidemiological study presented here uses an integrated environmental and biological monitoring approach. The aim is to assess in hospital nurses preparing and/or administering therapy to cancer patients the current level of occupational exposure to antineoplastic drugs, DNA and chromosome damage as cancer predictive effects, and the association between the two.

**Methods/Design:**

About 80 healthy non-smoking female nurses, who job it is to prepare or handle antineoplastic drugs, and a reference group of about 80 healthy non-smoking female nurses not occupationally exposed to chemicals will be examined simultaneously in a cross-sectional study. All the workers will be recruited from five hospitals in northern and central Italy after their informed consent has been obtained.

Evaluation of surface contamination and dermal exposure to antineoplastic drugs will be assessed by determining cyclophosphamide on selected surfaces (wipes) and on the exposed nurses' clothes (pads). The concentration of unmetabolized cyclophosphamide as a biomarker of internal dose will be measured in end-shift urine samples from exposed nurses.

Biomarkers of effect and susceptibility will be assessed in exposed and unexposed nurses: urinary concentration of 8-hydroxy-2-deoxyguanosine; DNA damage detected using the single-cell microgel electrophoresis (comet) assay in peripheral white blood cells; micronuclei and chromosome aberrations in peripheral blood lymphocytes. Genetic polymorphisms for enzymes involved in metabolic detoxification (i.e. glutathione *S*-transferases) will also be analysed.

Using standardized questionnaires, occupational exposure will be determined in exposed nurses only, whereas potential confounders (medicine consumption, lifestyle habits, diet and other non-occupational exposures) will be assessed in both groups of hospital workers.

Statistical analysis will be performed to ascertain the association between occupational exposure to antineoplastic drugs and biomarkers of DNA and chromosome damage, after taking into account the effects of individual genetic susceptibility, and the presence of confounding exposures.

**Discussion:**

The findings of the study will be useful in updating prevention procedures for handling antineoplastic drugs.

## Background

The occupational risk of environmental contamination during the storage, reconstitution, administration of antineoplastic drugs and the elimination of residues is well documented [1-4]. The chemical and physical properties of the drug, the quantity administered, the availability of personal and collective protection devices and the worker's skill determine the level of antiblastic contamination.

Several studies carried out at hospital units have shown detectable levels of cytotoxic agents in the air [5-7], on surfaces [8-15], on gloves [8,14], and on different parts of the body [7,8,16]. Biological monitoring methods have been developed to detect occupational exposure to antineoplastic agents [17]. The presence of these drugs in the urine of hospital personnel has been widely studied [7,9,18-20]. This has lead several organizations to develop guidelines or recommendations with the aim to improve safety during the handling of antineoplastic drugs and reduce risk of contamination in the workplace [21-23]. Based on these findings, guidelines have been also published in Italy [24].

Many anticancer agents have the potential to cause genetic alterations, which may lead to the development of cancer if they occur in proto-oncogenes or tumour-suppressor genes, which are involved in controlling cell growth or differentiation [25]. Accordingly, several antineoplastic drugs have been classified by the International Agency of Research on Cancer (IARC), on the basis of epidemiological reports, animal carcinogenicity data, as well as the outcomes of *in vitro *genotoxicity studies, as definite (Group 1), probable (Group 2A) or possible (Group 2B) human carcinogens [26-29].

Although health care workers are exposed to much lower doses than cancer patients are, low-dose exposure over long periods can have long-term health effects.

Several epidemiological studies have been conducted investigating the cancer risks of nurses exposed to antineoplastic drugs. Increased risks for leukaemia and breast cancer were reported by Skov et al. [30] and Gunnarsdottir et al. [31]. In a more recent research article, Ratner et al. [32] performed a cohort study among over 56,000 Canadian female nurses from British Columbia and concluded that subjects potentially exposed to antineoplastic drugs through their employment had an elevated risk of breast and rectal cancer.

Following environmental monitoring studies on contamination of workplaces from antineoplastic drugs, several biological monitoring studies have been performed. A number of studies indicate that antineoplastic drugs may cause increased genotoxic effects in pharmacists and nurses exposed in the workplace.

Undeger et al. [33] reported a significantly higher frequency of DNA damage - analysed using the alkaline single cell gel electrophoresis technique (comet assay) - in lymphocytes of nurses handling antiblastic drugs compared to unexposed controls; the DNA damage was, however, found to be significantly lower in nurses using compulsory personal protection equipment during their work. 8-hydroxy-2'-deoxyguanosine (8OHdG) - presumed to be an expression of oxidative damage to DNA - has never been used in assessing the mutagenic risk of occupational exposure to antineoplastic drugs.

Chromosome aberration (CA) frequencies in patients undergoing chemotherapy were significantly higher than in controls [34-37]. Increased CA frequencies have also been found in hospital personnel handling cytotoxic drugs [18,38-43]. Negative findings have also been reported, however [44-47]. In hospitals where nurses used inadequate safety cabinets when handling cytostatics, significantly elevated levels of CAs (as well as sister chromatid exchanges, SCEs, and unscheduled DNA-repair synthesis) were detected by Jakab et al. [48].

According to Kevekordes et al. [49], a malfunctioning safety hood resulted in a higher frequency of micronuclei (MN) and SCE in exposed nurses compared to matched controls. Kasuba et al. [50] found that the length of handling cytostatic drugs increased the frequency of MN, whereas no statistically significant difference was observed for SCE. In hospital pharmacy personnel adopting high standards of safety, Pilger et al. [51] observed that frequencies of MN and SCE were similar to those of controls, whereas small increases in these genetic end-points were found in accidental contamination events. Anwar et al. [43] found statistically significant increases in both CAs and MN in nurses handling cytostatic drugs. Hessel et al. [52] reported no association between antiblastic drugs in urine and MN frequency in lymphocytes of exposed hospital workers. Lastly, Maluf & Erdtmann [53], when comparing pharmacists and nurses exposed to antineoplastic drugs with unexposed controls, found no statistical difference for MN and dicentric bridge frequency, whereas the mean value of DNA migration detected by comet assay was significantly higher in the exposed group compared to the controls.

Several studies showed the influence of metabolic and DNA repair polymorphisms on biological indicators of genotoxic risk (urinary metabolites, protein and DNA adducts, citogenetic tests), which are commonly used in the biomonitoring of occupational exposure to antineoplastic agents [54]. There are however few studies on the influence of genetic polymorphisms of enzymes involved in DNA damage induced by occupational exposure to antineoplastic drugs [55,56].

In conclusion, some chemical studies have assessed occupational exposure to antineoplastic drugs, and some epidemiological investigations have detected various toxicologic effects in groups of exposed workers. To our knowledge, only very few researches have studied the same population by simultaneous assessment of exposure, biologic effects and genetic susceptibility [57].

The protocol of this molecular epidemiology study therefore presents an integrated chemical and biotoxicological approach for environmental and biological monitoring of exposure and cancer risks which will be implemented in a large number of healthy non-smoking female hospital nurses. This approach is based on monitoring procedures reported on the Italian guidelines [24] which includes, beside methods for preventing exposure to antineoplastic drugs, also monitoring recommendations. In particular, the guidelines provides guidance on the control of antineoplastic drug contaminations on surfaces and clothes by environmental monitoring (wipe and pad tests, respectively) and on the control of exposure by biological monitoring (concentrations of antineoplastic drugs in body fluids, usually urine), with both contamination and exposure depending on working practices and the frequency and adequacy of decontamination procedures. In this context, the advantage of biological monitoring is being able to measure the total uptake of antineoplastic drugs by all routes of exposure, however, testing is generally limited to one or very few agents that are considered as model compounds. In this study, monitoring of genotoxic risks we will performed by combining environmental and biological monitoring as above, with procedures for biological effect monitoring (urinary 8OHdG, DNA strand breakage and cytogenetic abnormalities in lymphocytes) as well as genetic susceptibility monitoring (metabolic polymorphisms).

## Study objectives

The aim of this study is to assess:

• the level of exposure to antineoplastic drugs (concentrations of substances in the working environment and on clothes; levels of biomarkers of exposure in end-shift urine) in nurses preparing and administering antineoplastic drugs;

• the profile of DNA and chromosome damage (cytogenetic and DNA primary alterations) and genetic polymorphisms in the same nurses preparing and/or administering therapy to cancer patients and in a control group of unexposed nurses;

• the association between exposure and genetic damage, taking into account the confounding effects of non-occupational exposures and the modulating effects of genetic polymorphisms.

Among the several biotoxicological tests that will be performed, the frequency of micronuclei (MN) and chromosome aberrations (CA) in peripheral lymphocytes are recognized to be predictors of cancer risks in human populations [58,59].

## Methods/Design

**Figure 1:**
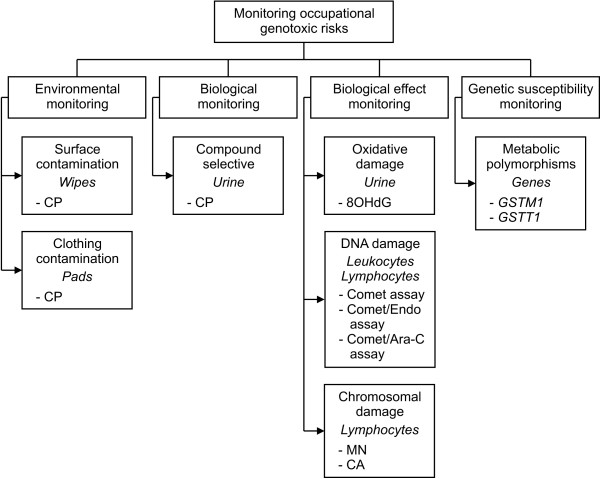
**Occupational genotoxic risks in healthcare workers handling antineoplastic drugs: scheme of the study protocol**. CP: cyclophosphamide; 8OHdG: 8-hydroxy-2'-deoxyguanosine; MN: micronuclei; CA: chromosome aberrations; GST: glutathione-*S*-transferase.

The proposed epidemiological study will be carried out following the integrated environmental and biological monitoring approach shown in Figure 1.

### Population recruitment

The study will be conducted on about 80 healthy non-smoking female nurses whose job it is to prepare and administer antineoplastic drugs. Workers will be recruited on a voluntary basis from five hospital departments in northern and central Italy. A reference group of about 80 healthy non-smoking female nurses - working in the same hospitals but not occupationally exposed to antineoplastic drugs, comparable for age and lifestyle habits - will be sampled and examined in parallel with the exposed group. Exclusion criteria will be male gender, active smoking, and radiography, radiotherapy or chemotherapy in the past 12 months.

An informed consent form will be signed by each worker included in the study.

### Sample collection

Work-shift urine samples will be collected from the workers in the investigated workplaces. Urine will be continuously collected 4 hours after the beginning until the end of the work-shift. During the same sampling session, end-shift blood samples will be collected taken by venipuncture and collected in heparinized or lithium EDTA vacuum tubes, for cytogenetic analyses or genotyping, respectively [60-62].

### Questionnaires

A questionnaire regarding the work environment will be designed to gather information from the ward sister on key characteristics of each hospital ward, namely: details of the preparation area (hood: type, discharge type, coverage plan, inlet and outlet filters, filters replacement and cleaning procedures), as well as conditions in the preparation and administration areas (appropriate space, exclusive use, access restrictions; presence of warning signs, dressing room, sinks, eyewash, shower; adequate lighting and ventilation, microclimate conditions; waterproof uniform intact floor and walls).

Trained interviewers will collect information from each subject by means of three other standardized questionnaires (A, B, and C).

Questionnaire A, which will be administered to both exposed and unexposed nurses, investigate personal details (age, height and weight), previous and present diseases, lifestyle habits (diet, passive smoking, alcohol and medicine consumption, physical activity and other leisure activities), and non-occupational exposures to mutagenic and carcinogenic agents, such as polycyclic aromatic hydrocarbons (PAH) present in diet (weekly consumption of charcoaled and/or smoked meat), indoor (use of wood or coal to heat the home) and outdoor environment (residence close to intense traffic and/or factories) [61,63].

Questionnaires B and C, which will be administered to exposed nurses only, investigate work experience and occupational exposures. Emphasis will be placed on gathering the following information:

• hospital ward, years of service, length of exposure for each task (preparation or administration of cytostatic drugs, patient care, waste disposal) performed during a typical work-shift;

• antineoplastic drug exposure: names of cytostatics most frequently handled in the previous 12 months (questionnaire B); names and quantities of cytostatics handled during the last two workshifts (questionnaire C);

• safety measures adopted by each worker (use of personal protection equipment, PPE; hand washing frequency; other).

### Environmental monitoring of exposure

Chemical analysis of the environmental samples will be carried out by determining the concentration of cyclophosphamide (CP), the marker of exposure to antineoplastic drugs according to established guidelines [24]. CP concentration will be determined in the work environment by using two techniques:

• surface removal technique (wipe-test);

• pad technique, to evaluate the contamination of exposed nurses' clothes.

#### Wipe test

The wipe sampling procedure will be performed according to the method of Sessink et al. [64], modified [65]. The technique involves cleaning a standard surface measuring 15 × 15 cm (225 cm^2^) thoroughly using a set of folded wipes (non-woven disposable material) wetted with 2 ml of 0.03 M NaOH solution. The standard sampling sites will be located on the hood surface (preparation site) or the drip surface (administration site). Blank wipes will also be tested. New gloves will be used for each collected wipe sample to prevent cross-contamination. The wipes will be put in test-tubes and transferred to the laboratory. Subsequently, 18 ml of 0.03 M NaOH solution will be added. The samples will be stirred for 10 minutes, sonicated for 30 minutes, centrifuged at 3000 rpm for 20 minutes and stored at -20°C until analysis.

The analysis will be carried out with GC-MS/MS. Briefly, a 1 ml of sample will be added with 100 μl of ifosfamide (IS) as internal standard. The solution will be put in a cartridge containing diatomaceous heart and the analytes eluted with 5 ml of ethyl acetate (twice). Organic layers will be combined and dried in a vacuum centrifuge. The residue will be dissolved in 100 μl of ethyl acetate and the CP and IS will be derivatized by adding 50 μl of trichloroacetic anhydride at 70°C for 30 minutes. The solvent will be evaporated in a vacuum centrifuge, and the residue will be dissolved in 50 μl of toluene and analysed in GC-MS/MS.

#### Pad test

The pad technique - widely used for monitoring xenobiotics [5] - will be used to evaluate contamination of clothes of nurses exposed to antineoplastic drugs. A pad of non-woven disposable material will be applied to clothes of the left forearm (non-dominant arm) of each subject during the working shift. The pad will be dampened with 1 ml of 0.03 M NaOH solution. The pads will be recovered at the end of the shift, placed in test-tubes and transferred to the laboratory, where they will be treated according to the procedure used for the wipe-tests.

On the whole, 1 wipe test (hood surface) and 1 pad test (left forearm) will be performed for each worker preparing antineoplastic drugs; 1 wipe test (drip surface) and 1 pad test (left forearm) will be carried out for each worker administering the drugs.

### Biological monitoring of exposure

To allow a better evaluation of exposure, a biomarker of internal dose (CP) will be measured in end-shift urine samples using an analytical method developed by Barbieri et al. [66]. Urine samples will be collected from the exposed workers during days that CP is handled.

The analysis will be performed by liquid chromatography coupled with a triple-quadrupole mass spectrometer equipped with an electrospray source (LC-ESI-MS/MS) after urine sample purification and concentration using solid phase extraction (SPE). Mass spectrometric detection increases sensitivity, and multiple-reaction-monitoring (MRM) acquisition mode leads to high specificity (i.e. the limit of detection is 0.04 μg/l urine for CP). Another aim of the study will be also to improve the sensitivity of the above method using a micro-HPLC, with lower flows (10 μl/min versus 200 μl/min for conventional HPLC) and smaller sample volumes (0.5 μl versus 20 μl).

### Biomarkers of DNA and chromosome damage

The biomarkers of DNA damage will be assessed in exposed and unexposed nurses.

#### Urinary concentration of 8OHdG

T he determination of urinary 8-hydroxy-2-deoxyguanosine (8OHdG) will be determined in both study groups, i.e. workers exposed and not exposed to antineoplastic drugs. The method, validated by Sabatini et al. [67], is based on high-performance capillary liquid chromatography coupled with an electrospray source with tandem mass spectrometric detection (micro-HPLC-ESI-MS/MS). Urine samples will be collected and extracted using Isolute Env+ SPE cartridges, and 0.5 μl of this extract will undergo micro-HPLC-ESI-MS/MS. MS/MS analysis will be conducted in positive ion mode and acquisition will be performed in MRM mode, selectively monitoring the typical transition of 8OHdG.

#### Comet Assay

To assess primary DNA damage under alkaline conditions, the single-cell microgel electrophoresis (comet) assay [68,69] will be performed on peripheral blood leukocytes of exposed and control nurses [60,70-72]. For slide preparation, 10 μl aliquots of heparinized blood will be mixed with 100 μl low-melting-point agarose (0.7% in PBS) and layered onto pre-treated conventional slides. After overnight lysis (+4°C) in alkaline buffer (pH 10) of cellular and nuclear membranes, the slides will be placed in a horizontal electrophoresis box, allowed to unwind for 20 min in an electrophoretic alkaline buffer (pH > 13) and then subjected to electrophoresis (+4°C) for 20 min by applying an electric field of 1 V/cm and adjusting the current to 300 mA. Lastly, the microgels will be neutralized and stained with ethidium bromide. To evaluate DNA damage, the slides will be examined using an epi-fluorescence microscope equipped with a high-sensitivity CCD (charge-coupled device) camera connected to a computerized image analysis system. Computerized imaging will be performed on coded slides using dedicated software which estimates damage parameters (i.e. tail length, tail intensity and tail moment) by comet profile [73]. Two hundred cells will be analysed for each subject (100 cells/slide, 2 slides per subject). Since the three research units examining DNA damage will use different computerized analysis systems, intercalibration tests will be performed to standardize the procedures used for DNA migration evaluation. For this purpose, the extent of DNA migration will also be evaluated by "visual scoring" based on visual classification of DNA damage.

#### Comet/Endo-III assay (DNA oxidative damage)

DNA oxidative damage will be evaluated in peripheral blood lymphocytes with a modification of the standard comet assay using enzymes of the excision repair system [74]. Endonuclease III (Endo-III) recognizes and cuts oxidized bases, mostly pyrimidines [75,76]. When this enzyme nicks DNA at sites of oxidatively damaged nucleotides, it creates single-strand breaks(SSB) detectable using the alkaline comet assay. For slide preparation, freshly collected white blood cells will be included in agarose microgels as described for the standard comet assay. After the lysis step, the microgels will be incubated with Endo-III for 60 min at 37°C. The slides will undergo alkaline electrophoresis (30 min unwinding and 20 min electrophoresis) and then stained as described for the standard comet assay. To evaluate DNA damage, the slides will be examined as described above for the standard procedure of the comet assay.

#### Comet/Ara-C assay

A modified protocol that uses a DNA repair inhibitor has been proposed as a means for increasing the sensitivity of the assay [77]. In particular, lymphocyte incubation with cytosine arabinoside (Ara-C) inhibits DNA re-synthesis during nucleotide excision repair and under standard experimental conditions transforms 'cryptic' lesions into SSB detectable with the alkaline comet assay. The evaluation of DNA damage to cells treated with Ara-C will be performed on peripheral blood lymphocytes isolated from whole blood samples using polysucrose density-gradient. The lymphocytes will be re-suspended in RPMI-1640 medium and cultured for 16 h in the presence or the absence of Ara-C (1 μg/ml) [78]. At the end of the culture time the cells will be washed and harvested by centrifugation, and the pellets will be mixed with low-melting-point agarose, subjected to alkaline lysis and electrophoresis, and stained and analysed as described for the standard comet assay.

#### Cytokinesis-block micronucleus test

All the subjects (exposed and controls) will be tested for the presence of MN in lymphocytes. Cell cultures will be set up by adding 0.3 ml of whole blood to 4.7 ml of RPMI-1640 medium supplemented with 20% foetal calf serum, 2 mM *L*-glutamine, 2% phytohaemoagglutinin and penicillin-streptomycin (100 IU/ml e 100 μg/ml, respectively). Whole blood cultures will be incubated for 72 h at 37°C, 5% CO_2 _[79]. To have binucleated cells, cytochalasin B (final concentration 3 μg/ml) will be added after 44 h [80]. The cells will then be collected by centrifugation, re-suspended in a pre-warmed hypotonic solution (75 mM KCl) for 15 min at 37°C and fixed in acetic acid - methanol (1:5 v:v). Air-dried preparations will be stained with 4% Giemsa. For cytogenetic analysis, a total of 1000 binucleated lymphocytes with preserved cytoplasm will be scored for each subject. MN evaluation will be based on standard criteria [81].

#### Chromosome aberration test

The entire population of enrolled subjects will also be tested for the presence of CA. Cell cultures will be set up by adding 0.5 ml of whole blood to 4.5 ml of RPMI-1640 medium supplemented with 10% foetal calf serum, 2% phytohaemoagglutinin and 1.5% penicillin-streptomycin. Whole blood will be cultured at 37°C, 5% CO2, following 90 minutes treatment with 0.2 μg/ml colcemid. 5-Bromodeoxyuridine will be added at a final concentration of 10 μg/ml. Cultures will be fixed at 48 h according to standard protocol [82]. Air-dried metaphase spreads will be stained according to the conventional unbanded Giemsa method. For cytogenetic analysis, an average of 100 well-spread metaphases per subject will be examined by optical microscopy. The analysis of CA will be performed only on cells with 46 chromosomes (+/- 1). CA will classified on the basis of standard criteria [83]. Both chromosome- and chromatid-type aberrations will be scored. Chromosome and chromatid breaks will be distinguished from gaps according to their break size and morphology.

### Biomarkers of genetic polymorphisms

Genetic polymorphisms for enzymes involved in metabolic detoxification (GSTM1 and GSTT1) will be analysed in exposed and unexposed nurses.

#### Genotype analysis

DNA from peripheral blood leucocyte (PBL) pellets will be isolated with a Promega Wizard genomic DNA purification kit (Promega, Italy). As described previously [84], the procedure will provide DNA free of RNA and protein contamination. A multiplex PCR method will be used to detect the presence or absence of the *GSTM1 *and *GSTT1 *genes, according to the protocol described previously [85]. This PCR method presents both *GSTM1*- and *GSTT1*-specific primer pairs in the same amplification mixture and includes a third primer pair for β-globin as an internal positive PCR control. The *GSTT1 *(480 bp), β-globin (285 bp), and *GSTM1 *(215 bp) amplification products will be resolved in an ethidium bromide-stained 2% agarose gel. The absence of the *GSTM1*- or *GSTT1*-specific fragment indicates the corresponding null genotype (*0/*0), whereas the β-globin-specific fragment confirms the presence of amplifiable DNA in the reaction mixture. Quality control measures will be adopted for validation of results using RT-PCR and blind repeat of 10% of samples.

### Statistical analysis

Statistical analysis will be performed to determine the association between occupational exposure to antineoplastic drugs (evaluated by environmental and biological monitoring) and biomarkers of DNA and chromosome damage, taking into consideration the effect of genetic polymorphisms, the safety procedures adopted, and confounding exposures of non-occupational origin.

The required sample size for the comparison of means from two independent samples was estimated assuming:

• the means and standard deviations reported in literature for DNA damage detected using the single-cell microgel electrophoresis (comet) assay in peripheral white blood cells, MN and CA in peripheral blood lymphocytes;

• a type I error rate of 0.05 and a power of 0.80.

Using the module "sampsi" of STATA 10 statistical software, the minimum sample size was of 47 exposed and 47 control nurses. The actual sample will be enlarged to include 80 individuals in each sample, to take also account of the number of predictors in the multiple regression analysis.

### Ethical approval

The study protocol has been firstly approved by the Ethics Committee of Health Institutions of Umbria Region (CEAS, Comitato Etico delle Aziende Sanitarie della Regione Umbria), Perugia and then by the other regional ethics committees (Comitato Etico dell'Azienda Ospedaliero-Universitaria di Bologna, Policlinico Sant'Orsola-Malpighi, Bologna; CEIOC, Comitato Etico Istituzioni Ospedaliere Cattoliche, Brescia; Comitato di Etica dell'Università degli Studi di Parma, Parma).

## Discussion

In a cross-sectional design such as ours, the prevalence of a disease is measured in relation to its determinants. Prevalence is a composite parameter, which depends on the incidence rate, the rate of cure, the fatality rate, and the duration of the disease. In addition, in studies of occupational epidemiology, health-selective turnover may further distort the information contained in the prevalence rate.

A follow-up of the study base - an incidence study - is better suited to solving etiologic problems than a cross-sectional one. The incidence of a disease is usually more informative than its prevalence, and incidence studies require a longitudinal design in which exposure and outcome are measured at different points in time.

Although cross-sectional designs have no time dimension, they can sometimes provide etiologic information. One example is the study of diseases with short or no latency, such as respiratory symptoms caused by irritant gases.

This study comprises short-term risk factors (names and quantities of cytostatics handled during the last two workshifts, collected on questionnaire C; CP in urine) and short-term response indicators (DNA strand breaks in polymorphonuclear leukocytes which have a lifetime of few hours to few days, as well as urinary 8OHdG). It should be noted that questionnaire C will be filled in the same day on which urine (for CP concentration) and blood (for comet assay) will be collected. We will therefore be able to determine whether there is an etiological association between occupational exposure to cytostatics and DNA damage.

This study also includes medium-term changes (cytogenetic abnormalities in lymphocytes which have a lifetime of weeks to years). AC and MN are of particular importance for prevention, since recent epidemiological studies have demonstrated the predictive value of carcinogenic risks from these two biomarkers [58,59].

The difference between exposed and unexposed nurses with regard to changes in short-term response (comet assay) could be less evident than that concerning medium-term effects (AC and MN). These results would indicate overall working conditions less satisfactory or safe in the past than in current conditions.

Any association between CP contamination and the use of PPE and number of preparations/day could potentially suggest useful information for prevention: an increase in health education if contamination is found to increase with relaxed rules of prevention, or a decrease in the workload if the number of applications is found to conflict with the level of prevention.

Both short-term and medium-term indicators of DNA damage are non-specific responses which depend on several risk factors of occupational and non-occupational origin. In order to prevent confounding, it has been decided to focus on one category of nurses, only female nurses declaring themselves to be long-life non-smokers. There are also other restrictions (see exclusion criteria in Methods). This choice requires the number of hospitals involved to be increased to 5 in order to achieve a sample size with sufficient statistical power. Restriction alone is a weak method for controlling confounding, and this can only be achieved by proper statistical analysis. Therefore, in order to control for non-occupational confounders, we have devised questionnaire A to gain information on a large number of potential risk factors.

## Competing interests

The authors declare that they have no competing interests.

## Authors' contributions

The authors contributed equally to this work. All authors have taken part in the academic discussions of the manuscript's content, in drafting the article and in revising it. All authors have approved the final version.

## Pre-publication history

The pre-publication history for this paper can be accessed here:

http://www.biomedcentral.com/1471-2458/11/195/prepub
